# COPAIBA OIL INFLUENCES VENTRAL HERNIA REPAIR WITH VICRYL^®^ MESH?

**DOI:** 10.1590/S0102-67202015000300010

**Published:** 2015

**Authors:** Edson Yuzur YASOJIMA, Renan Kleber Costa TEIXEIRA, Abdallah de Paula HOUAT, Felipe Lobato da Silva COSTA, Vitor Nagai YAMAKI, Denilson José Silva FEITOSA-JUNIOR, Carlos Augusto Moreira SILVA, Marcus Vinicius Henriques BRITO

**Affiliations:** (Experimental Surgery Laboratory, Faculty of Medicine, State University of Pará), Belém, PA, Brazil

**Keywords:** Surgical meshes, Wound healing, Rats

## Abstract

**Background:**

The use of meshes in hernia surgical repair promoted revolution in the surgical area; however, some difficulties had come, such as a large area of fibrosis, greater postoperative pain and risk of infection. The search for new substances that minimize these effects should be encouraged. Medicinal plants stand out due possible active ingredients that can act on these problems.

**Aim:**

To check the copaiba oil influence in the repair of abdominal defects in rats corrected with Vicryl^(c)^ mesh.

**Method:**

Twenty-four Wistar rats were submitted to an abdominal defect and corrected with Vicryl^(c)^ mesh. They were distributed into two groups: control and copaíba via gavage, administered for seven days after surgery. The analysis of the animals took place on 8, 15 and 22 postoperative days. It analyzed the amount of adhesions and microscopic analysis of the mesh.

**Results:**

There was no statistical difference regarding the amount of adhesions. All animals had signs of acute inflammation. In the control group, there were fewer macrophages in animals of the 8^th^ compared to other days and greater amount of necrosis on day 8 than on day 22. In the copaiba group, the number of gigantocytes increased compared to the days analyzed.

**Conclusion:**

Copaiba oil showed an improvement in the inflammatory response accelerating its beginning; however, did not affect the amount of abdominal adhesions or collagen fibers.

## Abstract (pt)

**Racional:**

A utilização de telas nas herniorrafias foi grande revolução na área cirúrgica; contudo, elas trouxeram algumas dificuldades, como grande área de fibrose, maior dor pós-operatória e risco de infecção. A busca por novas substâncias que minimizem esses efeitos deve ser estimulada. As plantas medicinais se destacam por apresentaram conjunto de princípios ativos que podem atuar em todos esses problemas.

**Objetivo:**

Verificar se o óleo de copaíba influência no reparo de defeitos abdominais em ratos corrigidos com tela de Vicryl^®^.

**Método:**

Vinte e quatro ratas Wistar foram submetidas a um defeito abdominal e corrigidos com tela de Vicryl^®^. Elas foram distribuídas em dois grupos: controle e copaíba via gavagem, administrada durante sete dias após a operação. A análise dos animais ocorreu nos dias 8, 15 e 22 de pós-operatório. Foi analisada a quantidade de aderências e feita análise microscópica da tela.

**Resultados:**

Não houve diferença estatística em relação à quantidade de aderências. Todos os animais tiveram sinais de inflamação aguda. No grupo controle, houve menor quantidade de macrófagos nos animais do dia 8 em relação aos demais dias e maior quantidade de necrose no dia 8 do que no dia 22. No grupo copaíba, o número de gigantócitos aumentou em relação aos dias analisados.

**Conclusão:**

O óleo de copaíba mostrou melhora na resposta inflamatória acelerando o seu início; contudo, não interferiu na quantidade de aderências abdominais ou fibras colágenas.

## INTRODUCTION

Hernia is defined as an abnormal organ or tissue protrusion through a weakness in the abdominal wall 8 . The main treatment for this condition is to perform hernia repair using tension-free techniques. The employment of surgical meshes in this procedure changed the relapse rates from 50-75% to 5-10% 3 4 .

The first report of the use of mesh in hernia repair was in 1958 by Usher et al., using polypropylene mesh 15 . This material remains the most common used in surgery; however, many types of surgical meshes are available with different materials, and with different sizes, density and elasticity 1 11 12 .

The use of mesh for hernia repair is associated with various side effects, such as increased postoperative pain, abdominal adhesions, fibrosis, and increased risk of infection due to placement of prosthesis 13 . Several therapies have been used to minimize these adverse effects without fully convincing results, for example anti-inflammatory that can relief pain, but interfering with the healing process and final abdominal tensile strength 14 18 .

Therefore, new therapies to minimize those effects are relevant and necessary. In this context, the use of medicinal plants has been reinforced, especially the copaiba oil ( *Copaifera sp* .). Derived from a tree (Copaifera) native of the Amazon region, it has proven anti-inflammatory, wound healing, and antibiotic effects; therefore, can improve multiple side effects of meshes usage 10 18 19 .

Previous experience of this authors group showed reduction in the quantity of adhesions and increase in the amount collagen fibers, based on inflammatory process modulation 19 . In this new study, was propose the use of the same oil, but applied after the surgery in order to verify if there is a potentiation of the anti-inflammatory and healing effects.

## METHODS

This research followed the rules of the Brazilian National Law for Animal Care (Law: 11.794/08) based on NIH guidelines, and followed the rules of Council for International Organization of Medical Sciences ethical code for animal experimentation. The project was previously approved by the animal committee at Pará State University. 

Twenty-four Wistar rats ( *Rattus norvegicus* ) aged about 120 days and with weight ranging from 200-250 g were included, having water and food provided ad libitum. They were divided into two groups with 12 animals: 1) control group (CG), treated with saline; 2) copaiba group (CopG), treated with copaiba oil, both administered by gavage from the 2^nd^ to 8^th^ postoperative days, at a dose of 0.63 ml/kg 19 .

These groups were subdivided into three subgroups (n=4) to assess the time of action of copaiba oil, 8^th^, 15^th^ and 22^nd^ postoperative days, when euthanasia of each subgroup was performed. 

All procedures were performed under anesthesia with ketamine hydrochloride (70 mg/kg) and xylazine hydrochloride (10 mg/kg), injected intraperitoneally. Once animals' anesthesia was confirmed, epilation of the abdominal region was performed, followed by antisepsis of the skin. Subsequently, a median 4 cm incision was performed on both sides for exposure of the aponeurotic muscle layer.

**FIGURE 1:**
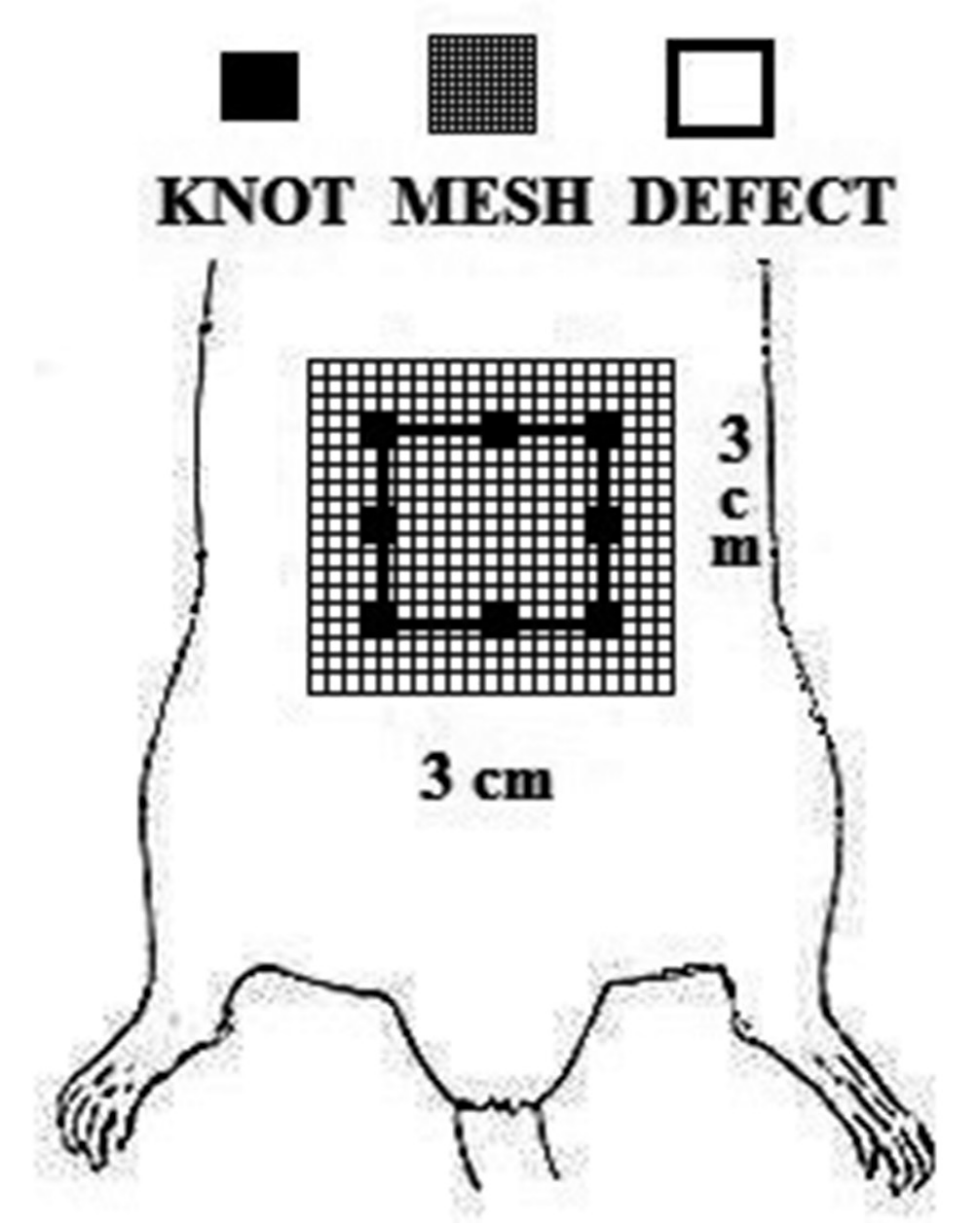
Surgical protocol

Immediately after, excision of the ventral part of the abdomen ( Figure 1 ), involving the aponeurotic muscle layer and the peritoneum with 2 cm in longitudinal axis and 2 cm in transversal in order to create a ventral defect in the aponeurotic muscle, was performed. This defect was corrected in all groups with the placement of Vicryl^(c)^ (polyglactin 910) mesh with 3 cm in longitudinal axis and 3 cm in transversal axis, attached at the edges with eight separated stitches (6-0 nylon thread), equidistant, with atraumatic needle and, with five semi-knots in each stitch, leaving the prosthesis margins over the anterior aponeurotic plane 19 .

After euthanasia, the site of surgery was assessed for presence or absence of incisional hernias, infections, dehiscence or fistulas and the number of adhesions, based on the semi-quantitative score of Diogo Filho et *al* . 5 that rate on 0=absence of adhesion; 1=one or two adhesions; 2=three to five adhesions; and 3=six or more adhesions. After this, scar tissues above and nearby meshes and the entire mesh was dissected for histological analysis. This fragment was stored in 10% buffered formaldehyde and used for histopathological analysis using of hematoxylin, eosin, and Masson's trichrome stains. 

In the hematoxyline/eosine stain, inflammatory response parameters were analyzed. Macrophage, lymphocyte and giant cell counting around mesh fragments; necrosis, fibrosis and granuloma area were classified as 0=absence; 1=mild; 2=moderate; and 3=intense 6 . Through Masson's trichrome stain were quantified amount and organization of collagen fibers, being classified as 0=absence; 1=mild; 2=moderate; and 3=intense 2 .

Normal distribution of data was confirmed using the Kolmogorov-Smirnov test. Results were analyzed by Kruskal-Wallis test. P-value less than 0.05 was considered statistically significant for all tests.

## RESULTS

Through the macroscopic analysis, no infections, dehiscences or fistulas were observed. However, all animals had incision hernias and formation of adherences between the meshes and the abdominal organs, showing no difference between the control and copaiba groups in the three scheduled periods ( Table 1 ).

**TABLE 1:** Average number of adhesions between the mesh and the abdominal organs

Group	TIME	
	8 days	15 days	22 days
CG	1.00 ±0.82	1.25 ±0.50	2.25 ±0.96
CopG	0.50 ±0.58	1.50 ±1.00	1.50±0.58

p=0.11 (Kruskal-Wallis)

All animals showed acute inflammatory response ( Table 2 ), characterized by the presence of edema, vascular congestion and infiltrated with predominance of neutrophils. Regarding to the quantity of other immune system components, there was a smaller amount of macrophages in the CG in relation to the CopG (p=0.03) in the 8^th^ postoperatory day. There was a higher amount of giant cells in the CopG than in the CG in the 22^nd^ postoperatory day (p=0.03). The amount of lymphocytes was similar in both groups (p=0.22).

**TABLE 2:** Average of inflammatory cell counting surrounding mesh fragments.

Group	TIME	
	8 days	15 days	22 days	Macrophages
CG	0.00±0.00	2.50±0.57	2.50±1.00
CopG	1.75±0.50	1.75±0.95	1.25±0.50	Gigant cells
CG	8.33±4.93	9.00±4.90	9.75±4.44
CopG	1.25±1.25	7.75±4.03	22.25±8.54	Lymphocytes
CG	1.33±0.57	2.25±0.96	2.25±0.96
CopG	1.00±0.00	1.00±0.00	1.25±0.50

Macrophages: CG 8 days x CopG 8 days - p=0.03; gigant cells: CG 8 days x CopG 8 days and CG 22 days x CopG 22 days - p=0.03; Lymphocytes: CG x CopG - p=0.31

Granulomas formed around the meshes were histologically characterized as a foreign body-type granuloma composed of macrophages and giant cells around each mesh fragment. The intensity (size) of the granuloma in CG was 2.18±0.60 and in CopG was 1.75±0.45 (p=0.06).

Necrotic areas were not identified macroscopically; however, microscopic zones of liquefaction necrosis were identified close to the meshes. In the CG and CopG, zones of necrosis were generally of medium intensity with no significant difference between the groups (p=0.94). The formation of fibrosis areas was similar in CG and CopG (p=0.93) presenting intensity 1.36±0.67 in GC and 1.41±0.51 in CopG. 

In the Masson's trichrome stain, the amount of collagen fibers in the groups was measured. The results are described in Table 3 ; there was no significant difference regarding their amount or distribution.

**TABLE 3:** Average of amount and organization of collagen fibers

Group	TIME	
	8 days	15 days	22 days	Amount
CG	1,50±0,57	1,25±0,50	1,66±1,15
CopG	1,00±0,81	1,75±1,50	1,75±0,50	Organization
CG	2,00±0,00	0,50±0,57	1,00±1,00
CopG	1,25±0,50	1,50±0,57	1,50±0,57

p>0.05 (Kruskal-Wallis)

## DISCUSSION

The search for factors that can improve the healing process and reduce its side effects dates back from the Hippocratic era; at present, it is not available therapeutic method that can fully control the harmful effects of this process. The search for new drugs should be encouraged for better control of the wound inflammation which in many cases cause surgical complications 13 14 18 .

The medicinal plants are major sources of active principles that can be used in the pharmaceutical industry. In the context of hernioplasty with meshes, the copaiba oil's healing and anti-inflammatory effects may improve the inflammatory process and minimize complications without harming the abdominal wound 10 19 .

The absence of fistulas, dehiscence and infections show that the Copaiba oil did not influence in the healing process 18 . The presence of hernias in both groups demonstrates that the studied mesh (Vicryl^(c))^ failed to keep the abdominal strength; it is believed that this occurred because of the manipulation of the animal for gavage administration which probably stretched the abdominal region.

Yasojima et al. 19 evidenced reduction in the amount of abdominal adhesions when offered the copaiba oil prior to the hernia repair. In this study there was a reduction; however, this was not statistically significant, showing that prophylactic use of copaiba oil had better effects in reducing adhesions. Additional doses may probably extend the oil effects and be able to reduce the amount of adhesions.

The inflammatory response was similar in both groups; however, there was an earlier beginning on the copaíba group, showing higher levels of macrophages on the 8^th^ day after surgery. These represent the primary cell type responsible for the control and regulation of healing, prior to migration and replication of fibroblasts. This earlier appearance might suggest the mechanism for the improvement of the healing process using this oil 7 9 17 .

In regard to the amount of gigant cells, it was higher in the copaiba group after 22 days postoperatively, however with a smaller granuloma size. Therefore, the copaiba oil might eventually inactivate this cell group, increasing its amount, but without impair the healing process.

The size of necrosis and fibrosis areas were similar in both groups, representing the tissue repair process. This data demonstrates that, even with an earlier initiation of the inflammatory response, there was no major impact on the repair process. It shows a low therapeutic effect of the oil in the administered dose.

In relationship to collagen fibers, the amount and organization were similar in both groups. Although with effects on the modulation of the inflammatory response, the copaiba oil did not show a change in the repair of the healing process. There was almost no effect when compared to other studies where this oil was supplied prophylactically 16 19 .

## CONCLUSION

Oral administration of copaiba oil modulated inflammatory response and did not impair tissue healing; however, it affected the amount of abdominal adhesions or collagen fibers.
